# Supplementary material for: Temporal patterns and risk factors of diarrheal comorbidity among children aged < 5 years in rural western Kenya: Evidence from three consecutive enteric studies, 2008–2024
Source: PLOS Glob Public Health. 2026 Feb 13;6(2):e0005532. doi: 10.1371/journal.pgph.0005532 (PMC12904429; doi:10.1371/journal.pgph.0005532)
Supplement: S1 Table — (DOCX) [file pgph.0005532.s001.docx]

S1 Table. Diarrheal comorbidities based on Integrated Management of Childhood Illness (IMCI) case definitions and clinician diagnosis

| **IMCI Clinical symptom** | **IMCI Description** | **Questions used to determine if the clinical symptom was present** |
| --- | --- | --- |
| Uncomplicated SAM | WHZ < -3 or  MUAC < 115mm (11.5cm) and  Able to finish a ready-to-use therapeutic food (RUTF) | **WHZ and MUAC:**  Study clinician took anthropometric measurements at the time of enrollment including:   - weight in kg (with and without the caretaker for children under 2 months) - mid-upper arm circumference (MUAC) in cm (measured 3 times for accuracy) - height in cm (measured 3 times for accuracy)   The child’s weight-for-height z-score (WHZ) was estimated using the WHO z-score method and the median of the three weight measurements. The median of the three MUAC measurements was estimated.  **RUTF:**  There were no questions at the time of enrollment which captured information about the child’s ability to finish RUTF. |
| Complicated SAM | WHZ < -3 or  MUAC < 115mm (11.5 cm) or  bipedal edema and  medical complication (  any general danger sign or  any other severe classification or pneumonia with chest indrawing) or  not able to finish RUTF (6 months or older) or  breastfeeding problem (less than 6 months) | **WHZ and MUAC:**  Described previously for uncomplicated SAM  **Bipedal edema:**  Presence of bipedal edema was reported by study clinician.  **Any general danger sign:**  The caregiver was asked if the child had been unable to drink, experienced convulsing, loss of consciousness, decreased activity or lethargy since the diarrheal illness began. The caregiver was also asked if the child had experienced any vomiting or if they were currently (at the time of enrollment) drinking poorly/not able to drink or lethargic/had loss of consciousness. The child being lethargic or unconscious could also be reported by the study clinician.  **Any other severe classification:**  The following severe classification and outlined in this table:   - Severe or very severe pneumonia - Very severe Febrile Disease - Uncomplicated severe Acute Malnutrition   A child with at least two of the following was considered to have severe dehydration:   - Caregiver reported a child drinking much less than usual or being unable to drink - Study clinician recorded the child was lethargic or unconscious - Study clinician recorded the eyes were sunken (confirmed with the caregiver that the eyes were more sunken than usual) - Study clinician recorded a very slow (> 2 seconds) skin pinch return rate   Neither mastoiditis or severe anemia were recorded but could be noted by study clinician as the diagnosis at the time of hospital discharge.  **Pneumonia with chest indrawing:**  Pneumonia is outlined in this table and those who met this definition due to chest indrawing, as opposed to fast breathing, are considered here.  **RUTF/Breastfeeding:**  There were no questions at the time of enrollment which captured information about the child’s ability to finish RUTF or difficulties with breastfeeding. |
| Pneumonia | Cough or difficulty breathing + chest indrawing or fast breathing  Fast breathing is defined as 50 or more breaths per minute for a child 2 months up to 12 months or 40 or more breaths per minute for a child 12 months up to 5 years. | **Cough/difficulty breathing:**  A cough or difficulty breathing, since the diarrheal illness began, could be reported by the caregiver at the time of enrollment. A cough could also be recorded by study clinician as a diagnosis at hospital discharge.  **Chest indrawing:**  Chest indrawing was recorded by study clinician  **Fast breathing:**  Respiratory rate per minute was recorded twice by study clinician at enrollment and the average of these two measurements was used in conjunction wit the child’s calculated age (based on provided DOB) to determine if fast breathing was present.  Pneumonia can also be indicated as a diagnosis at hospital discharge and would be recorded by study clinician. |
| Severe or very severe pneumonia | Cough or difficulty breathing +  a general danger sign or  stridor in a calm child | **Cough/difficulty breathing:**  Described previously for pneumonia  **Any general danger sign:**  Described previously for complicated SAM  **Stridor in a calm child:**  There were no questions asked at the time of enrollment which captured information about stridor. |
| Acute febrile disease | Regardless of malaria risk:  Child fells hot or has a temperature of 37.5$℃$ or above +  any general danger sign or  a stiff neck | **Fever:**  A fever, since the diarrheal illness began, could be reported by the caregiver. Study clinician also measured to child’s axilliary temperature at the time of enrollment.  **Any general danger sign:**  Described previously for complicated SAM  **Stiff neck:**  There were no questions asked at the time of enrollment which captured information about stiff neck. |
| **Other clinical symptoms** | | |
| Bacterial infection | | An invasive bacterial infection diagnosis recorded by study clinician. |
| Meningitis | | A diagnosis of meningitis recorded by study clinician. |
| Respiratory illness (Non-pneumonia) | | A diagnosis of influenza like illness or acute respiratory illness recorded by study clinician. |
| Anemia | | A diagnosis of anemia recorded by study clinician. |
| Malaria | | A diagnosis of malaria recorded by study clinician. |
| Stunting | | A height-for-age z-score (HAZ) below -2 standard deviations (SD) from the WHO Child Growth Standards median at enrolment. |
| Wasting | | A weight-for-height z-score (WHZ) between >-3 and <-2 SD from the WHO Child Growth Standards median at enrolment. Mutually exclusive with SAM |

Uncomplicated SAM and complicated SAM combined to create SAM;

Pneumonia and severe pneumonia combined to create pneumonia variable.
