# Supplementary material for: Temporal patterns and risk factors of diarrheal comorbidity among children aged < 5 years in rural western Kenya: Evidence from three consecutive enteric studies, 2008–2024
Source: PLOS Glob Public Health. 2026 Feb 13;6(2):e0005532. doi: 10.1371/journal.pgph.0005532 (PMC12904429; doi:10.1371/journal.pgph.0005532)
Supplement: S2 Table — (DOCX) [file pgph.0005532.s002.docx]

S2 Table. Factors associated with the number of comorbidities among children aged 0–59 months presenting with moderate-to-severe diarrhea in Western Kenya, 2008–2024, including rotavirus vaccination status

| Variable | Category | Bivariate | | Multivariable |
| --- | --- | --- | --- | --- |
|  |  | **Unadjusted IRR [95% CI]** | **P-value** | **Adjusted IRR [95% CI]** |
| Age Categories | 0_11 m | Ref |  | Ref |
|  | 12_23 m | **1.05 [1.01-1.09]** | **0.011** | 1.03 [0.98-1.08] |
|  | 24_59 m | **1.07 [1.02-1.12]** | **0.002** | **1.07 [1.02-1.13]** |
| Gender | Female | **0.95 [0.91-0.98]** | **0.003** | **0.95 [0.91-0.99]** |
| Caregiver education less than primary school | Yes | **1.14 [1.10-1.19]** | **<0.001** | **1.08 [1.04-1.13]** |
| Improved water | Unimproved | Ref |  | Ref |
|  | Improved | **0.94 [0.91-0.97]** | **<0.001** | **0.96 [0.92-0.99]** |
| Improved sanitation | Unimproved | Ref |  | Ref |
|  | Improved | **0.94 [0.91-0.97]** | **0.001** | 1.01 [0.97-1.06] |
| Respiratory Rate categories^β^ | Normal | Ref |  | Ref |
|  | Low | **0.86 [0.78-0.94]** | **0.002** | 0.91 [0.80-1.03] |
|  | High | **1.27 [1.22-1.33]** | **<0.001** | **1.21 [1.14-1.28]** |
| Max no. of watery diarrhea episodes in a day, n (%) | ≤ 6 | Ref |  | Ref |
|  | ≥7 | **0.91 [0.88-0.94]** | **<0.001** | 1.00 [0.95-1.04] |
| Pre-enrolment diarrhea days |  | 1.01 [0.99-1.02] | 0.211 | - |
| Experienced vomiting |  | **1.24 [1.19-1.29]** | **<0.001** | **1.25 [1.19-1.32]** |
| Dehydration | None | Ref |  | Ref |
|  | Severe | **1.46 [1.32-1.62]** | **<0.001** | **1.34 [1.18-1.51]** |
|  | Some | **1.28 [1.15-1.42]** | **<0.001** | **1.23 [1.10-1.39]** |
| Vesikari score Categories^¥^ | Mild | Ref |  | Ref |
|  | Moderate | **1.27 [1.17-1.38]** | **<0.001** | - |
|  | Severe | **1.53 [1.41-1.66]** | **<0.001** | - |
| Fully vaccinated^§^ | No | Ref |  | Ref |
|  | Yes | 0.96 [0.92-1.00] | 0.062 | 1.01 [0.96-1.06] |
|  | Unknown^ŧ^ | **1.11 [1.06-1.16]** | **<0.001** | 1.06 [0.99-1.14] |
| PCV10 vaccine | Unvaccinated | Ref |  | Ref |
|  | Vaccinated ( ≥ 1) | 0.90 [0.78-1.03] | 0.116 | 0.93 [0.80-1.07] |
|  | Unknown^ŧ^ | 1.09 [0.95-1.25] | 0.232 | 0.94 [0.81-1.10] |
| Rotavirus Vaccine | Prevaccine | Ref |  | Ref |
|  | Vaccinated ( ≥ 1) | **0.80 [0.77-0.83]** | **<0.001** | 1.01 [0.90-1.14] |
|  | Unvaccinated | **0.80 [0.75-0.86]** | **<0.001** | 1.01 [0.88-1.15] |
|  | Unknown^ŧ^ | **0.90 [0.83-0.97]** | **0.005** | 1.14 [0.98-1.33] |
| Rainfall (mm) tercile | Tercile 1:13.92-<82.49 | Ref |  | Ref |
|  | Tercile 2:82.49-<158.30 | 1.01 [0.96-1.05] | 0.814 | 1.02 [0.96-1.09] |
|  | Tercile 3:158.30-<353.40 | 0.99 [0.94-1.03] | 0.537 | 1.02 [0.95-1.10] |
| Rainfall 3-month lag (mm) tercile | Tercile 1:13.92-<82.49 | Ref |  | Ref |
|  | Tercile 2:82.49-<158.30 | 1.05 [1.00-1.11] | 0.052 | **1.07 [1.01-1.14]** |
|  | Tercile 3:158.30-<353.40 | 1.04 [0.98-1.09] | 0.188 | **1.08 [1.02-1.16]** |
| LSTD (°C) tercile | Tercile 1:26.36-<30.09 | Ref |  | Ref |
|  | Tercile 2:30.09-<32.96 | 0.98 [0.94-1.02] | 0.390 | 0.98 [0.93-1.03] |
|  | Tercile 3:32.96-<41.38 | 0.97 [0.92-1.01] | 0.135 | 0.93 [0.86-1.00] |
| LSTD 3-month lag (°C) tercile | Tercile 1:26.36-<30.09 | Ref |  | Ref |
|  | Tercile 2:30.09-<32.96 | 0.99 [0.93-1.04] | 0.645 | 1.03 [0.98-1.08] |
|  | Tercile 3:32.96-<41.38 | 1.05 [1.00-1.11] | 0.064 | **1.10 [1.04-1.16]** |
| year | year | **0.97 [0.96-0.97]** | **<0.001** | **0.97 [0.96-0.99]** |

*IRR-Incidence Rate Ratios

AFI-Acute Febrile illness; LSTD- Land Surface Temperature – Daytime

^β^Cutoffs for respiratory rate based on the Pediatric Advanced Life Support (PALS) guidelines.

¥- Modified 17-point Vesikari score

^§-^Full vaccinated- 1 dose of BCG, 3 doses of pentavalent and polio, and 1 dose of measles

^ŧ-^Unknown vaccination card not available
