# Supplementary material for: Temporal patterns and risk factors of diarrheal comorbidity among children aged < 5 years in rural western Kenya: Evidence from three consecutive enteric studies, 2008–2024
Source: PLOS Glob Public Health. 2026 Feb 13;6(2):e0005532. doi: 10.1371/journal.pgph.0005532 (PMC12904429; doi:10.1371/journal.pgph.0005532)
Supplement: Attachment — Submitted filename: Point_by_Point Response_12.01.2026.docx [file pgph.0005532.s004.docx]

**Point-by-point response to Editor/Reviewer Comments**

We sincerely appreciate the invaluable comments and suggestions provided by the editor and the reviewers, which we believe have contributed in improving the quality of this work. Below, we present a point-by-point response to each of the remarks (in italic), clearly highlighting the revisions made in the manuscript (with line numbers (**highlighted in bold**) referenced in the marked version) and offering clarifications where needed.

**Editorial Comments to Author:**

Journal Requirements:

**Comment 1:** Please send a completed 'Competing Interests' statement, including any COIs declared by your co-authors. If you have no competing interests to declare, please state "The authors have declared that no competing interests exist".

***Response 1****: Thank you for this comment. A 'Competing Interests' statement is included in Lines* ***524-525****.*

**Comment 2:** Please amend your detailed Financial Disclosure statement. This is published with the article. It must therefore be completed in full sentences and contain the exact wording you wish to be published. State the initials, alongside each funding source, of each author to receive each grant.

***Response 2****: We appreciate this comment. The financial disclosure statement has been updated to include initials of authors for each funding source.*

**Comment 3:** We have noticed that you have uploaded Supporting Information files, but you have not included a list of legends. Please add a full list of legends for your Supporting Information files after the references list.

***Response 3****: Thank you for this comment. A full list of legends for the Supporting Information files has been included after the references list (Lines* ***710-717****).*

**Comment 4:** If the reviewer comments include a recommendation to cite specific previously published works, please review and evaluate these publications to determine whether they are relevant and should be cited. There is no requirement to cite these works unless the editor has indicated otherwise. 
***Response 4:*** *We appreciate this comment. No specific recommendations for additional references were provided by the reviewers.*

**Additional Editor Comments (if provided):**

**Comment 5:** Thank you for submitting your manuscript entitled “Temporal Patterns and Risk Factors of Diarrheal Comorbidity among Children aged < 5 years in Rural Western Kenya: Evidence from Three Consecutive Enteric Studies─2008-2024”to PLOS Global Public Health.
The manuscript has been evaluated by external reviewers, whose detailed comments are provided below. Both reviewers acknowledge the importance of the topic and note that the manuscript is generally well written and well organized. The pooled analysis of data from GEMS, VIDA, and EFGH is considered a valuable contribution with relevance for diarrheal disease control and management in children under five years of age. However, several substantive concerns have been raised that must be addressed before the manuscript can be considered further.

***Response 5:*** *We appreciate comments from the reviewers. Their feedback was valuable, and we have incorporated the suggested changes into the manuscript, as outlined below.*

**Reviewer Comments to Author:

Reviewer 1:**

**Comment 1:** P124: Both GEMS and VIDA enrolled children under 5 while EFGH enrolled children aged 6-35 months. Please clarify whether this difference in age distribution or missing 0-5 months and 36-59 months would affect the analysis, especially growth-related factors.

***Response 1:*** *Thank you for this comment. We acknowledge that differences in age distribution across studies may influence the results and therefore conducted a sensitivity analysis restricted to children aged 6–35 months. The findings are largely consistent with the primary analysis. Additional findings included an association between PCV10 vaccination and a 16% lower number of comorbidities compared with unvaccinated children and higher comorbidity counts associated with three-month lagged land surface temperature in terciles 2 and 3 ( See Table 4). The relevant sections have been updated in the methods (Lines* ***192-194****) and results (Lines* ***341-346****).*

**Comment 2:** P137: Please clarify whether “bacterial infection” excluded diarrhea associated bacterial infection?

***Response 2:*** *Thanks. “bacterial infection” refers to Other invasive bacterial infection based on clinician diagnosis. This has been updated in the methods in Line* ***145****.*

**Comment 3:** P138: Would add an Oxford comma after “stunting”.

***Response 3:*** *Thank you. This has been updated accordingly.*

**Comment 4:** Figure 4: I would suggest to remove the numbers at the bottom.

***Response 4:*** *Thank you for the comment. Figure 4 has been updated accordingly.*

**Comment 5:** Table S1: As comorbidities were the focus of this work, I would suggest to add more details for “other clinical symptoms” regarding case definitions.

***Response 5:*** *We appreciate this comment. The “other clinical symptoms” refer to diagnoses made by study clinicians during participant evaluation. These diagnoses were based on clinical history and physical examination, as no additional diagnostic tests were performed to confirm these conditions.*

**Reviewer 2:**
**Comment 1:** I don't see a clear statement on whether rotavirus vaccination was assessed in the analyses. It is plausible that rotavirus vaccination may impact the risk of the comorbidities examined here, in particular severe acute malnutrition. Please provide some further information regarding how that was handled. Please also indicate if vaccine status (receipt of core vaccines, being fully vaccinated) was assessed in the analyses? I think some discussion of the impact of a major vaccine against diarrheal disease is warranted when assessing the risk factors for diarrheal comorbidities.

***Response 1:*** *Thank you for this comment. We have updated the analyses to include full vaccination status and PCV10 vaccination status in the primary analysis (Table 3) and in the analysis restricted to children aged 6–35 months (Table 4). An additional sensitivity analysis also includes rotavirus vaccination status and models calendar year as a continuous variable (Table S2), as calendar year and rotavirus vaccination status were perfectly confounded when year was modeled categorically. The Methods (Lines* ***153-164; 190-199****), Results (Lines* ***232-237; 313-315; 358-369****), and Discussion (Lines* ***410-414; 425-428; 465-472****) sections have been updated accordingly.*

**Comment 2:** I'm missing the utility of the climate data that is presented here. Very little reference is made in the discussion section to the possible role it may have as risk factor for diarrheal comorbidities. While climate metrics are important risk factors for disease burden shifts, they were not clearly identified as a risk factor here and the authors may wish to reconsider including it in this paper.

***Response 2:*** *We appreciate this comment. We have rerun all analyses, including the primary analysis, the analysis restricted to children aged 6–35 months, and the sensitivity analysis incorporating rotavirus vaccination status. In these updated models, the climate variables, particularly three-month lagged rainfall and LSTD, were statistically significant. The Results (Lines* ***319-321; 344-346****) and Discussion (Lines* ***472-477****) sections have been updated accordingly.*

Introduction:
**Comment 3:** Lines 82-24: Consider adding a sentence regarding the wider availability and use of Oral Rehydration Solution (ORS) as a relevant public health intervention

***Response 3:*** *Thank you for the comment. This has been updated accordingly in Lines* ***78-79.***

Methods:
**Comment 4:** Please include in lines 100-104 when Kenya introduced rotavirus vaccine and coverage rates.

***Response 4:*** *Thank you for this comment on rotavirus vaccine introduction and coverage. This has been updated in Lines* ***103-113.***

**Comment 5:** Did you collect data on vaccination status? Do you have information on rotavirus vaccination status for those children eligible to receive it in VIDA or EFGH?

***Response 5:*** *Thank you for this comment. Immunization data for GEMS and VIDA have been obtained from the HDSS, whereas EFGH collected comprehensive vaccination data directly. Accordingly, vaccination variables, including full vaccination status, PCV10, and rotavirus vaccination status, have now been incorporated into the re-analysis.*

**Comment 6:** Can you please clarify at what level did you collect climate covariates? Were these collected for all of Western Kenya, for Siaya County or for each individual participant’s address?
Results:

***Response 6:*** *We appreciate this comment on climate data. The monthly climate data were extracted using defined bounding boxes representing the study catchment area (Lines* ***170-171****).*

**Comment 7** Line 250, please provide the peaks of AFI you are referring to from Table 2 in the text.

***Response 7:*** *Thank you for the comment. This has been updated accordingly in Lines* ***288-289.***

**Comment 8:** Table 3 the rain and LSTD variables should be more clearly specified so that the table stands independently. I assume that Lag 1 is one-month lag, 2-month lag and 3 month lag respectively but this could be made clearer in the table. This table is also rather cumbersome and it may benefit from reducing the rain and LSTD variables to present only the LSTD 3 month lag category.

***Response 8:*** *Thank you for this comment. The climate variables have been updated accordingly in Tables 3, 4 and S2.*

Discussion
**Comment 9:** The authors provide valid explanations for the impact rotavirus vaccine and PCV10 have had on the reduction of viral diarrhea and on pneumonia cases respectively. They also provide a good explanation in lines 335-337 on the persistent and plausibly increasing circulation of other viral respiratory pathogens. Is it possible that some of the observed increase in respiratory illness comorbidities may also be due to evolving diagnostic options and awareness in the region since the 2009 influenza pandemic and COVID pandemic. Were changes made to ICMI criteria to expand diagnoses of non-pneumonia respiratory illness during the time frame of their study?

***Response 9:*** *Thank for this comment. We agree that revisions to IMCI guidelines in 2014 that refined pneumonia classification and emphasized objective assessment of hypoxaemia using pulse oximetry may have also contributed to the surge due reclassification of milder respiratory cases (Lines 425-428). See below a summary of changes to IMCI during the study period:* ***Between 2008 and 2024, IMCI guidelines for respiratory illness transitioned from a three-tier to a simplified two-tier classification system, notably reclassifying chest indrawing as non-severe pneumonia suitable for outpatient management. This shift was accompanied by the universal adoption of oral Amoxicillin Dispersible Tablets as the first-line antibiotic, replacing older regimens like Cotrimoxazole. Furthermore, the routine integration of pulse oximetry enhanced diagnostic accuracy by establishing hypoxemia (SpO2<90%) as a primary indicator for severe disease.***

Figure Resubmissions:

**Comment1:** While revising your submission, we strongly recommend that you use PLOS’s NAAS tool (<https://ngplosjournals.pagemajik.ai/artanalysis>) to test your figure files. NAAS can convert your figure files to the TIFF file type and meet basic requirements (such as print size, resolution), or provide you with a report on issues that do not meet our requirements and that NAAS cannot fix.

After uploading your figures to PLOS’s NAAS tool - <https://ngplosjournals.pagemajik.ai/artanalysis>, NAAS will process the files provided and display the results in the "Uploaded Files" section of the page as the processing is complete. If the uploaded figures meet our requirements (or NAAS is able to fix the files to meet our requirements), the figure will be marked as "fixed" above. If NAAS is unable to fix the files, a red "failed" label will appear above. When NAAS has confirmed that the figure files meet our requirements, please download the file via the download option, and include these NAAS processed figure files when submitting your revised manuscript.

***Response 1:*** *Thank you for this comment. All figures have been tested by NAAS and meet journal requirements.*
